# Vaccination With a Single Consensus Envelope Protein Ectodomain Sequence Administered in a Heterologous Regimen Induces Tetravalent Immune Responses and Protection Against Dengue Viruses in Mice

**DOI:** 10.3389/fmicb.2019.01113

**Published:** 2019-05-10

**Authors:** Ran Wang, Xiaoyan Zheng, Jin Sun, Kaihao Feng, Na Gao, Dongying Fan, Hui Chen, Xia Jin, Jing An

**Affiliations:** ^1^Department of Microbiology and Parasitology, School of Basic Medical Sciences, Capital Medical University, Beijing, China; ^2^Beijing Tropical Medicine Research Institute, Beijing Friendship Hospital, The Second Clinical Medical College of Capital Medical University, Beijing, China; ^3^Viral Disease and Vaccine Translational Research Unit, CAS Key Laboratory of Molecular Virology and Immunology, Institute Pasteur of Shanghai, Chinese Academy of Sciences, Shanghai, China; ^4^Center of Epilepsy, Beijing Institute for Brain Disorders, Beijing, China

**Keywords:** dengue virus, tetravalent vaccine, envelope, E80, consensus sequence, prime-boost

## Abstract

The development of a safe and effective tetravalent dengue vaccine that elicits protection against all dengue virus (DENV) serotypes is urgently needed. The consensus sequence of the ectodomain of envelope (E) protein of DENV (cE80) has been examined as an immunogen previously. In the current study, a cE80 DNA (D) vaccine was constructed and evaluated in conjunction with the cE80 protein (P) vaccine to examine whether both vaccines used together can further improve the immune responses. The cE80 DNA vaccine was administrated using either a homologous (DNA alone, DDD) or heterologous (DNA prime-protein boost: DDP or DPP) regimen, and evaluated for immunogenicity and protective efficacy in mice. Among the three DNA-based immunization regimens tested, DDP immunization is the optimal immunization regimen that elicited the greatest systemic immune response and conferred protection against all four DENV serotypes. This work provides innovative ideas for the development of consensus E-based dengue vaccines and the testing of optimal immunization regimens.

## Introduction

Dengue viruses (DENVs) belong to the genus *Flavivirus* of the family *Flaviviridae*, which includes several other major human pathogens such as Zika virus, Japanese encephalitis virus (JEV), and yellow fever virus (YFV) (Lazear and Diamond, 2016). Four distinct but closely related serotypes of DENV (DENV1–4) are the cause of a wide range of clinical presentations including dengue fever, dengue hemorrhagic fever, and dengue shock syndrome. Dengue is one of the most prevalent mosquito-borne viral diseases in tropical and subtropical countries with an estimated 390 million annual cases (Miksanek and Wolford, 2017). In the past 50 years, incidence of dengue has increased 30-fold, with expanded transmission from urban to rural settings (Stanaway et al., 2016; Aggarwal and Garg, 2017), making it a significant international public health problem.

As the most cost-effective public health tool, vaccination is indispensable for addressing the global burden of dengue, and thus the development of vaccine against dengue is urgently needed. Various dengue tetravalent vaccine candidates are currently in clinical and preclinical stage of development (Kirkpatrick et al., 2016; Sirivichayakul et al., 2016; Saez-Llorens et al., 2017; Whitehead et al., 2017), based mostly on combining four antigens representative of four DENV serotypes into a single tetravalent formulation. The CYD-TDV was the first and only licensed tetravalent dengue vaccine approved in thirteen dengue epidemic countries (Ferguson et al., 2016; Henein et al., 2017). It is comprised of four monovalent dengue vaccines, each of which is made by replacing genes encoding the precursor membrane and the envelope (E) proteins of each DENV serotype with the corresponding genes on the YFV-17D backbone. A recent study reanalyzed data from three efficacy trials of the CYD-TDV vaccine, and discovered that despite the vaccine conferred protection against severe dengue and hospitalization for dengue seropositive subjects for up to 5 years, but the same vaccine put dengue naïve individuals at a higher risk of infection and disease (Sridhar et al., 2018). These new data prompted a change in the vaccine label by the vaccine maker and a reappraisal of the CYD-TDV vaccination strategy by WHO Report (2018); Strategic Advisory Group of Experts (Wilder-Smith et al., 2019). An experimental tetravalent dengue vaccine, TV003, showed some promise during clinical testing. This vaccine was initially developed by the National Institutes of Allergy and Infectious Disease of the United Stated, and then licensed to the company Takeda. In phase I and II studies conducted in dengue-endemic and non-endemic countries, TV003 elicited robust neutralizing antibody (nAb) responses to all four DENV serotypes, and it was well-tolerated in all subjects (George et al., 2015; Rupp et al., 2015; Saez-Llorens et al., 2018). Currently, TV003 is being tested in a phase III clinical trial in Brazil (Whitehead et al., 2017). Other experimental dengue vaccines that are also under development have recently been reviewed (de Soarez et al., 2019).

It is generally believed that non-neutralizing cross-reactive antibody response induced by a certain DENV serotype may increase the risk of developing severe disease upon a secondary infection by a different DENV serotype due to antibody-dependent enhancement (ADE). Therefore, it is important for developing tetravalent vaccines that are capable of inducing balanced immune responses and broad protection against all four DENV serotypes simultaneously. Instead of using four separate components, a single vaccine with representative epitopes or consensus sequences from multiple strains, and serotypes of DENV may be a novel vaccine strategy needing exploration.

The developments in bioinformatics and computational approaches have brought versatility in vaccine design. For instance, the epitope-based vaccine design has been applied to a variety of pathogens in which immune responses could not be induced by conventional vaccines (Sette et al., 2001; Sette and Fikes, 2003). Such consensus sequence-based vaccine strategy had been applied to develop immunogens to elicit broadly reactive, cross-reactive, and protective responses against influenza A virus, human immunodeficiency virus (HIV), DENV, and other genetically complex pathogens (Carter et al., 2016; Meyerhoff et al., 2017; Sun et al., 2017; Wang et al., 2017). Many studies have demonstrated that consensus predictions could outperform single sequence determination method in vaccine design (Mallios, 2003; Moutaftsi et al., 2006; Yan et al., 2007). Importantly, not only conserved regions but also variable sites could be incorporated in the consensus sequences, and such design does not seem to alter structural and functional properties of target protein on a given location (Schein et al., 2012).

The E glycoprotein of DENV has been considered as an important antigen for dengue vaccine development, as it is the major component on the surface of DENV and the primary target for nAb (Trent, 1977; Beltramello et al., 2010). The E protein and its ectodomain (an N-terminal 80% truncated at amino 395, E80) are rich in serotype-specific neutralizing and cross-reactive epitopes, and they have been applied widely in diagnostics and vaccine development (Jarvi et al., 2013). To achieve as much coverage as possible of all E80 of four DENV serotypes, it is logical to construct a recombinant product composed of critical neutralizing epitopes of each serotype of DENVs. The rivet of epitopes from highly conserved regions of virus by using consensus sequence is a reasonable option for targeting different serotypes of DENV. Thus, we have previously designed a vaccine based on the most conserved regions of E protein ectodomain, named it consensus-based E80 (cE80), and then assessed its immunogenicity *in vivo* (Sun et al., 2017). Our results showed that immunization with three doses of the cE80 recombinant protein vaccine was capable of eliciting both Th1 and Th2 responses, and nAb responses to all four DENV serotypes in mice (Sun et al., 2017). In order to trigger broader immune responses, especially cellular immune responses, and to improve the potential protective efficacy of cE80 candidate vaccine, a new DNA vaccine expressing the cE80 was constructed, and tested in conjunction with the cE80 protein in the current study using either homologous DNA immunization regimen or heterologous DNA prime-protein boost regimens. Our results demonstrated that among the three DNA-base immunization regimens, two DNA prime plus one cE80 protein boost regimen evoked the strongest humoral and cellular immune responses to all four DENV serotypes, and conferred protection in mice against infection by each of the four serotypes of dengue viruses. This work provides new insights into the development of tetravalent dengue vaccines.

## Materials and Methods

### Cells, Viruses, and Mice

Vero cells were cultured in minimal essential medium (Gibco, United States) supplemented with 5% fetal bovine serum (FBS, Gibco, United States) at 37°C. C6/36 cells were cultured in RPMI-1640 medium (Gibco, United States) supplemented with 10% FBS at 28°C. All cells were cultivated under a humidified atmosphere of 5% CO_2_.

The DENV1 (Hawaii strain), the DENV2 (TR1751 strain), the DENV3 (H87 strain), and the DENV4 (H241 strain) were propagated in C6/36 cells and stored in a –80°C freezer. DENV particles were harvested from culture supernatant of C6/36 cells that had been infected by DENV, concentrated by 8% polyethylene glycol precipitation, and then purified from clarified extracts by ultracentrifugation.

Female BALB/c mice (6-week-old) were purchased from Beijing Vital River Laboratory Animal Technology Co., Ltd.

### Ethics Statement

The animal experiments were performed according to “Regulations for the Administration of Affairs Concerning Experimental Animals” which is the national guidelines for the care and use of animals in scientific research. All experimental procedures were approved by the Institutional Animal Care and Use Committee of Capital Medical University, China.

### *cE80* Sequence

The gene encoding consensus sequence cE80 [cE80(max)] was *in silico* calculated and synthesized as described previously (Sun et al., 2017). The cE80 sequence has 87.41–94.50% nucleotide homology and 73.33–88.25% amino acid homology with E80 sequences of the four representative DENV serotypes (Supplementary Table S1). The matching of cE80 with E80 of each serotype of DENVs at predicted amino acid level was shown in Supplementary Figure S1A.

### Construction and Purification of Recombinant Plasmid pV-cE80 and cE80 Protein

To construct a recombinant plasmid expressing the cE80 protein, the Kozak sequence and signal sequence from vesicular stomatitis virus glycoprotein (MKCLLYLAFLFIGVNC) was added upstream to the *cE80* DNA sequence (Supplementary Text S1). *Eco*RI and *Not*I sites were inserted upstream of the translation start codon and downstream of the termination codon, respectively. Finally, the synthetic *cE80* sequence was subcloned in between *Eco*RI and *Not*I sites of pVAX1 vector (hereinafter, pV), and named as pV-cE80.

Baculovirus-expressed cE80 protein used in immunization was constructed and purified as described previously (Sun et al., 2017).

### Immunization Schedule

**FIGURE 1:**
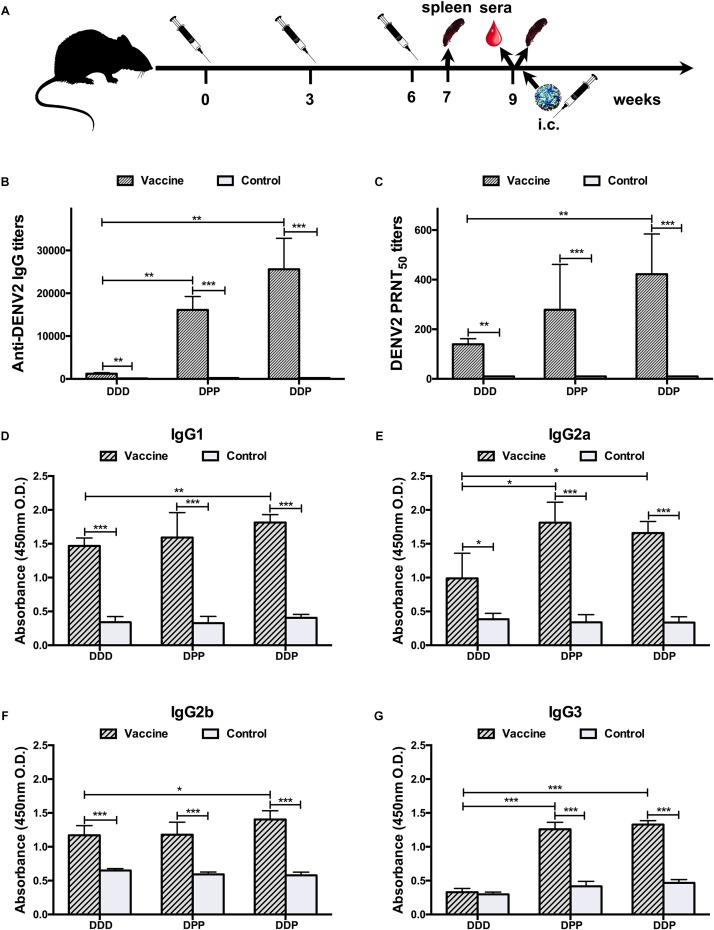
Immunization schedule and humoral immune response to DENV2 induced by different immunization regimens. **(A)** Immunization, sampling, and challenge timeline. **(B)** Anti-DENV2 IgG antibody titers. **(C)** Anti-DENV2 nAb titers in serum samples (*n* = 8) and data represent the GMT + SD. **(D–G)** Distribution of serum DENV2-specific IgG subclass responses, **(D)** IgG1; **(E)** IgG2a; **(F)** IgG2b; and **(G)** IgG3. The levels of IgG subclass were expressed as 450 nm OD. Data were expressed as means + SD (*n* = 8). ^∗^*P* < 0.05, ^∗∗^*P* < 0.01, ^∗∗∗^*P* < 0.001.

Mice were divided into groups receiving either homologous or heterologous prime-boost vaccinations (Supplementary Table S2). As shown in Figure 1A, mice in all groups were immunized three times at 3-week intervals. For the homologous immunization regimen, mice were immunized with 50 μg pV-cE80 in 50 μl of normal saline by intramuscular (i.m.) injection into the anterior tibialis muscle with *in vivo* electroporation (EP) as described previously (Wang et al., 2018), and this group was designated as DDD. The control mice were administrated with an equal quantity of pV.

For the heterologous prime-boost immunization regimens, mice were either inoculated i.m. once with 50 μg pV-cE80 by *in vivo* EP method and followed by boosted subcutaneously (s.c.) twice with 10 μg cE80 protein emulsified with 10 μl of Alhydrogel^®^ adjuvant (DPP), or primed with pV-cE80 twice and followed by boost with the cE80 protein once (DDP). The two corresponding control groups were given i.m. once/twice with 50 μg pV by *in vivo* EP method and followed by s.c. twice/once with an equal volume of adjuvant.

### Enzyme-Linked Immunosorbent Assay (ELISA)

To detect DENV-specific IgG antibodies and their subsets IgG1, IgG2a, IgG2b, and IgG3, each well of the 96-well plates was coated with 2 μg purified viral particles from DENV1, or DENV2, or DENV3, or DENV4; plates were then blocked with 3% bovine serum albumin; ELISA was performed according to a reported method (Wang et al., 2018). Sera from immunized mice were twofold serially diluted in PBS (from 1:100 to 1:204,800), DENV-specific antibodies were detected with goat-anti-mouse secondary antibodies (1:4,000, HRP coupled, Abcam, United States) and substrate solution of tetramethylbenzidine. The optical density (OD) at 450 nm was measured using an ELISA reader (Thermo Fisher Scientific, United States). The reciprocal of the highest dilution which yields an OD value that is greater than one half of the OD value of corresponding control at 1:100 dilution, was recorded as the end-point titer of IgG antibody.

For the detection of IgG subclasses, sera from immunized mice (at 1:100 dilution) were used as the primary antibodies, and the levels of IgG subclasses were expressed as OD value.

### Plaque Reduction Neutralization Test (PRNT)

Anti-DENVs nAb titers were measured using PRNT as described previously (Wang et al., 2018). DENV1 (Hawaii strain), DENV2 (TR1751 strain), DENV3 (H87 strain), and DENV4 (H241 strain) were used in this test. After heat inactivation at 56°C for 30 min, twofold serially diluted sera (from 1:5 to 1:1,280) were mixed 1:1 with DENV suspension containing 50 plaque-forming units (PFU), and incubated at 37°C for 1 h. The mixture was transferred to a Vero cell monolayer in a 24-well plate, and incubated at 37°C for another 1 h. The infected Vero cells were washed, then overlaid with MEM containing 1.2% methylcellulose. Plaques were stained and counted after 6–8 days of incubation at 37°C. The reciprocal highest serum dilution that corresponded to a 50% reduction of the average number of plaques on the virus infection only wells was determined as the neutralizing titer PRNT_50_.

### CD8^+^ T Cell Response in Splenocytes Measured by Flow Cytometry

All antibodies and other staining reagents were purchased from BD Biosciences, United States. After red blood cell lysis, splenocytes were blocked with rat anti-mouse CD16/CD32 monoclonal antibody, and then stimulated at 1.5 × 10^6^/ml with 1 μg purified DENV2 particles for 6 h. The cells were subsequently incubated with hamster anti-mouse CD3e-FITC, rat anti-mouse CD8-APC-H7, rat anti-mouse CD44-APC, and rat anti-mouse CD62L-PE. Finally, samples were analyzed on a DxFLEX flow cytometer (Beckman Coulter, United States) using CytExpert software (version 2.0), the complete gating strategy was shown in Supplementary Figure S2.

### Enzyme-Linked Immunospot (ELISPOT) Assays

Splenocyte-produced IL-4 and IFN-γ were determined using ELISPOT kits (BD, United States) as previously described (Wang et al., 2018). In brief, splenocytes isolated from immunized mice were aliquoted at 3 × 10^5^/well into 96-well filtration plates (Millipore, United States) pre-coated with capture antibodies and stimulated with 5 μg/well purified DENV particles for 60 h at 37°C. After incubation with biotinylated detection antibodies and streptavidin-HRP, respectively, the spots were visualized by adding 3-amino-9-ethylcarbazole substrate and then counted with an ELISPOT reader (CTL, United States) and analyzed by ImmunoSpot software (version 5.1).

### DENVs Challenge

To investigate the protective efficacy of vaccine candidates, mice were challenged 3 weeks after the last vaccination, intracerebrally (i.c.) with either 1 × 10^5^ PFU of DENV1 (Hawaii strain), or 200 PFU of DENV2 (Tr1751 strain), or 1 × 10^7^ PFU of DENV3 (H87 strain), or 1 × 10^5^ PFU of DENV4 (H241 strain), individually. The body weight change in individual mouse and survival rate were monitored daily for 12 days.

### Statistical Analysis

Statistical analyses were performed using SPSS version 17.0 (SPSS Inc., United States). The sequence alignments and homology were analyzed with DNASTAR software (Version 7.1, United States). Differences of mean body weight changes between groups were analyzed with repeated measures analysis of variance (ANOVA). Kaplan-Meier survival curves were plotted and evaluated statistically by Log-rank test. Comparisons between groups were analyzed using one-way ANOVA. The results were presented as means +/± standard deviation (SD), and the difference between means is considered significant if ^∗^*P* < 0.05, very significant if ^∗∗^*P* < 0.01, and extremely significant if ^∗∗∗^*P* < 0.001.

## Results

### Exploration of Optimal Immunization Strategy

To determine which vaccination regimen can elicit the most effective immune responses with cE80 DNA and cE80 protein vaccines, we first examined their antigenicity. Specific fluorescent signal was observed in the cytoplasm of transiently transfected cells, indicating that plasmid-expressed cE80 protein could be recognized by antibodies to four serotypes of DENV *in vitro*, and it could be used in subsequent experiments (Supplementary Figure S1B). We then compared three immunization strategies, including thrice DNA (DDD), DNA prime twice plus protein boost once (DDP), or DNA prime once plus protein boost twice (DPP) (Figure 1A and Supplementary Table S2). The humoral and cellular immune responses, cytokine responses, and protective efficacy against DENV2 were determined first to establish the optimal experimental conditions.

Three weeks after the last immunization, in the DDD vaccine group, the geometric mean titer (GMT) of IgG against DENV2 was 1:1,213 [95% confidence interval (CI), 1:877–1:1,703]; whereas the GMT in the control group was 1:131 (95% CI, 1:95–1:185, *P* < 0.01, Figure 1B). Next, the neutralizing capacity of DENV2-specific antibody was measured by PRNT. A GMT of anti-DENV2 nAb reached 1:139 (95% CI, 1:107–1:184) following the DDD regimen; none of the control pV-administrated mice showed detectable nAb activity (less than 1:10, *P* < 0.01, Figure 1C). These data demonstrated that the DDD regimen can induce DENV2-specific humoral immune responses in mice.

Mice immunized via heterologous prime-boost regimens (DPP and DDP) induced significantly higher IgG titers. The GMTs were 1:16,127 (95% CI, 1:11,874–1:22,210) and 1:25,600 (95% CI, 1:16,906–1:39,794) in the DPP and DDP regimens, respectively, which were 13- and 21-folds higher than that of the homologous DDD immunization group (*P* < 0.01, Figure 1B). A similar result of higher nAb titers was also observed. The sera nAb PRNT titers were 1:278 (95% CI, 1:133–1:637) and 1:422 (95% CI, 1:251–1:740) in the DPP and DDP regimens, respectively, which were 2–3-fold greater than that in the DDD regimen.

To further characterize the specific profile of IgG antibodies induced by different vaccination regimens, DENV2-specific IgG1, IgG2a, IgG2b, and IgG3 in immune sera were analyzed by ELISA (Figure 1D–G). In mice, it is known that IgG1 production is assisted by a Th2 response, whereas IgG2a, IgG2b, and IgG3 production is helped by a Th1 response (Germann et al., 1995), and thus by analyzing the antibody subclass distribution, we can deduce which T cell subset was activated with different vaccines. Our results showed that the DDD vaccination regimen mainly elicited IgG1, IgG2a, and IgG2b antibodies, but not the IgG3 subclass. In comparison, the heterologous regimens, DPP and DDP, evoked anti-DENV2 antibodies of all these subclasses, including IgG1, IgG2a, IgG2b, and IgG3. These data indicated that heterologous vaccination could induce better IgG diversification than homologous regimens, implying a better T cell activation.

Taking together, the above data showed that among all vaccination regimens tested, mice immunized with the DDP regimen developed the strongest anti-DENV2 humoral immune responses, the homologous DDD regimen induced the weakest, and the DPP performed in between.

**FIGURE 2:**
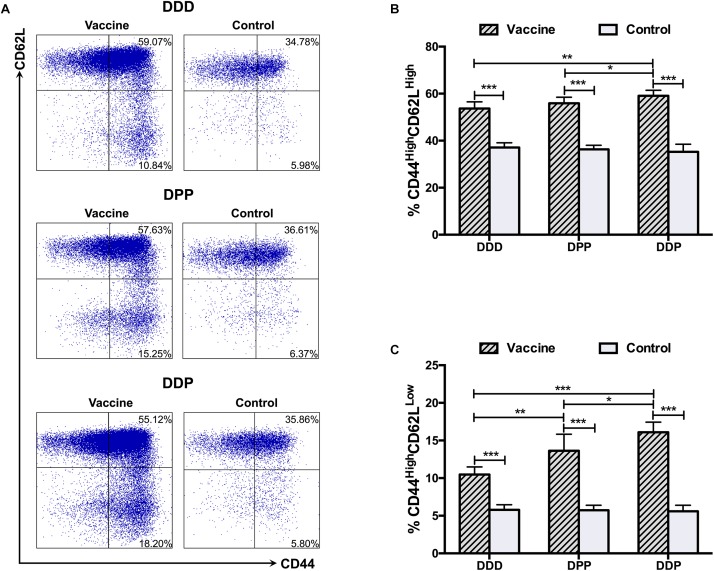
CD8^+^ T cell responses against DENV2 elicited by different immunization regimens. **(A)** Representative expression of CD44^High^ T cells (gate on CD3^+^ CD8^+^ T cells). Quantification of the frequency of **(B)** CD44^High^ CD62L^High^ and **(C)** CD44^High^ CD62L^Low^ cells among CD8^+^ T cells. Data were expressed as mean + SD (*n* = 8). ^∗^*P* < 0.05, ^∗∗^*P* < 0.01, ^∗∗∗^*P* < 0.001.

CD8^+^ T cell responses were analyzed 1 week after the third vaccination. We first investigated central (CD44^High^ CD62L^High^) and effector (CD44^High^ CD62L^Low^) memory T cells. Upon stimulation with DENV2 antigen, cells from mice received all three immunization regimens showed robust central and effector memory T cells responses. Notably, the highest proportion of both central (Figure 2A,B) and effector memory T cells (Figure 2A,C) was observed in the DDP regimen, and they were significantly higher than those in the DDD and DPP regimens. The DPP regimen also showed a higher proportion of effector memory T cells population, but not central memory T cells, than the DDD regimen (Figure 2B,C). These results suggested that heterologous vaccinations, especially the DDP regimen, effectively induced CD8^+^ T cell expansion.

**FIGURE 3:**
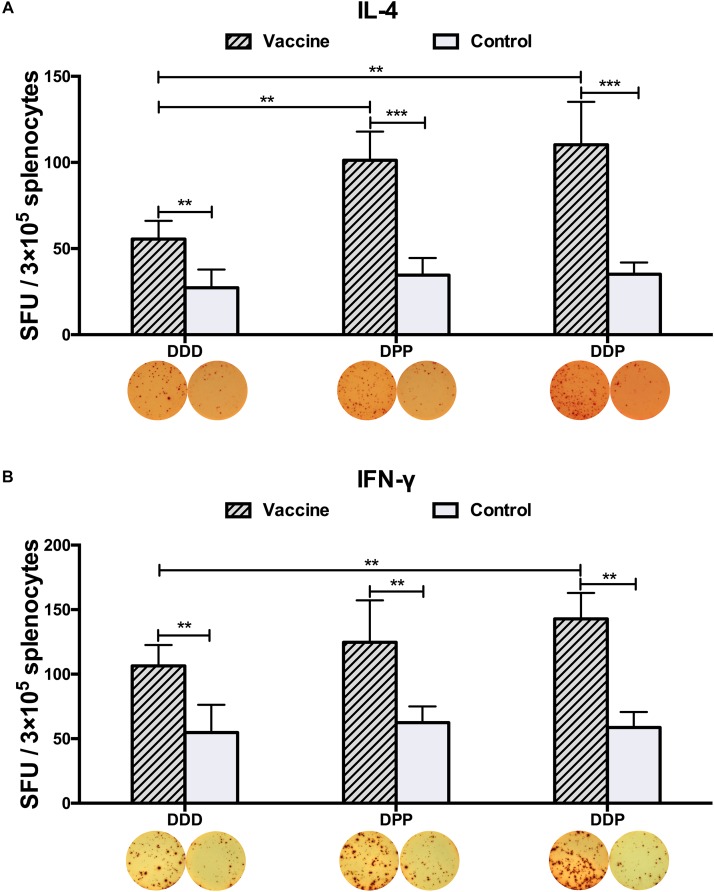
DENV2-specific cytokine-secreting splenocytes in different immunization groups. Cytokine-secreting splenocytes were determined after stimulation with purified DENV2 particles. The positive splenocytes were counted and expressed as the mean SFU/3 × 10^5^ cells. **(A)** IL-4-positve and **(B)** IFN-γ-positive splenocytes with representative ELISPOT images, respectively. Data were expressed as mean + SD (*n* = 8). ^∗∗^*P* < 0.01, ^∗∗∗^*P* < 0.001.

To examine whether these vaccines could activate CD4^+^ T cells equally, 3 weeks after the final immunization, we measured the generation of Th1- and Th2-associated cytokines. Upon stimulated with purified DENV2 particles, IFN-γ and IL-4 levels, as representative indications of Th1 and Th2 cell responses, were measured by ELISPOT assay. Mice vaccinated with all three immunization regimens had significantly triggered DENV2-specific IL-4 and IFN-γ responses as compared to controls (Figure 3A,B). The DPP regimen induced more IL-4 than the DDD regimen (Figure 3A), and the DDP regimen elicited the highest levels of both IFN-γ and IL-4 responses (Figure 3A,B), both were significantly higher than that in the DDD regimen (*P* < 0.05). These results again indicated that the heterologous DDP regimen was more effective at inducing both Th1 and Th2 cytokine responses than the other regimens.

**FIGURE 4:**
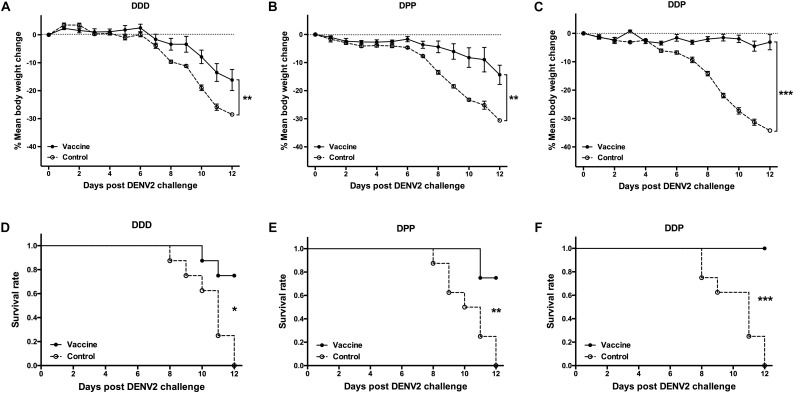
Protective efficacy against DENV2 challenge in different immunization regimens. Mice immunized with different regimens were challenged i.c. with DENV2 and monitored daily for body weight and survival rate for 12 consecutive days (*n* = 8). **(A–C)** Percentage changes of body weight from day 0 were determined as 100 × (weight post-challenge)/(weight pre-challenge). Data were expressed as mean ± SD, **(D–F)** The survival rate shown as the percentage of survivors. ^∗^*P* < 0.05, ^∗∗^*P* < 0.01, ^∗∗∗^*P* < 0.001.

To examine whether vaccine induced immune responses were associated with protection, 3 weeks after the final vaccination, each mouse was challenged i.c. with 200 PFU of DENV2 and the protective effect was evaluated. During the observation period, mice in all control groups showed 28–34% body weight loss (Figure 4A–C) and none of them survived the viral challenge (Figure 4D,E). In contrast, there was only a 16% of the mean body weight loss in the DDD group, significant less than controls (*P* < 0.01, Figure 4A); and 75% survived after viral challenge (*P* < 0.05, DDD vs. control, Figure 4D). The DPP group showed even less daily body weight loss (14%) than controls (*P* < 0.01, Figure 4B) and also a survival rate of 75% (*P* < 0.01, Figure 4E). Of note, the DDP vaccination regimen protected 100% of all mice (*P* < 0.001, Figure 4F), which showed no obvious body weight loss (*P* < 0.001, Figure 4C). Thus, these results showed that, among three DNA-based immunization regimens, the heterologous DDP prime-boost regimen is the optimal strategy because it conferred the most effective protection, with concurrent induction of the highest nAb level, activated CD8^+^ T cell, and elicited both Th1 and Th2 responses.

### Comprehensive Evaluation for Tetravalent Immune Responses of cE80 With the DDP Regimen

Having established the optimal vaccination strategy with a single dengue serotype, DENV2, we wanted to explore whether using the same DDP prime-boost regimen could induce tetravalent immune responses against all four dengue serotypes.

**FIGURE 5:**
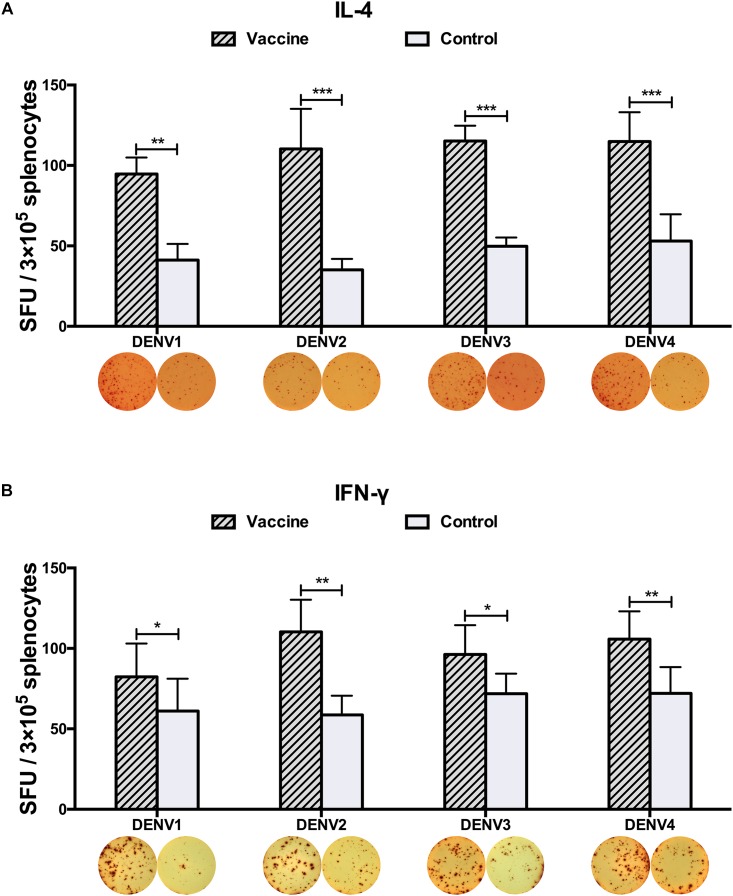
DENV1–4-specific cytokine-secreting splenocytes elicited by the DDP immunization regimen. Cytokine-secreting splenocytes were determined after stimulation with purified DENV1–4 particles, individually. The positive splenocytes were counted and expressed as the mean SFU/3 × 10^5^ cells. **(A)** IL-4-positve and **(B)** IFN-γ-positive splenocytes with representative ELISPOT images, respectively. Data were expressed as mean + SD (*n* = 8). ^∗^*P* < 0.05, ^∗∗^*P* < 0.01, ^∗∗∗^*P* < 0.001.

**FIGURE 6:**
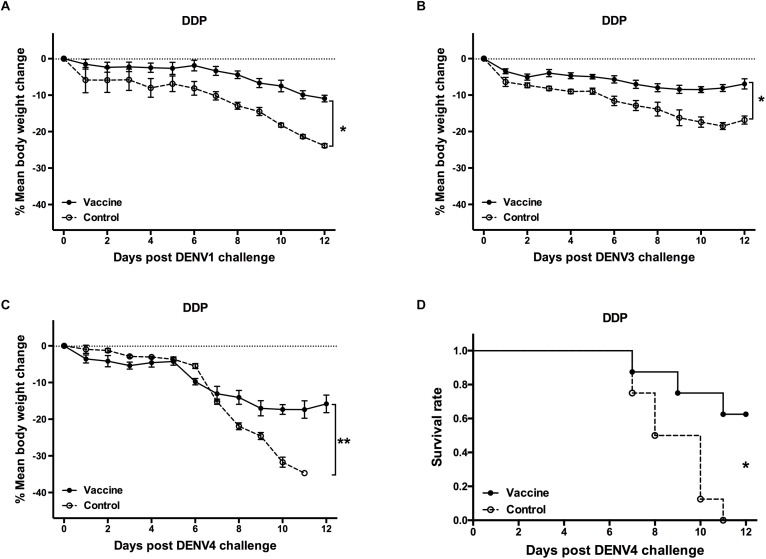
Protective efficacy against DENV1, DENV3, and DENV4 induced by the DDP immunization regimen. Mice immunized with the DDP regimen were challenged i.c. with DENV1, DENV3, and DENV4 and monitored daily for 12 days (*n* = 8). Percentage changes of body weight from day 0 after challenge with **(A)** DENV1, **(B)** DENV3, and **(C)** DENV4. The percentage was determined as 100 × (weight post-challenge)/(weight pre-challenge). Data were expressed as mean ± SD. **(D)** The survival rate post challenge with DENV4 was shown as the percentage of survivors. ^∗^*P* < 0.05, ^∗∗^*P* < 0.01.

Three weeks after the last vaccination using the cE80 vaccines with the DDP regimen, considerable IgG responses were generated to DENV1 and DENV2, with GMTs of 1:16,890 (95% CI, 1:6,432–1:32,176), and 1:25,600 (95% CI, 1:16,906–1:39,794), respectively; robust responses to DENV3 and DENV4 were also induced, with titers of 1:6,400 (95% CI, 1:6,400–1:6,400) and 1:4,850 (95% CI, 1:1,847–1:9,240), respectively (Table 1). Similarly, tetravalent neutralizing antibodies were generated by the cE80 vaccines against all four DENV serotypes, with PRNT_50_ titers of 1:160 (95% CI, 1:38–1:358) to DENV1, 1:422 (95% CI, 1:251–1:740) to DENV2, 1:106 (95% CI, 1:40–1:201) to DENV3, and 1:61 (95% CI, 1:23–1:116) to DENV4 (Table 1). We next examined T cell responses induced by the DDP regimen. Three weeks after the final immunization, splenocytes were isolated and stimulated with purified DENV1–4 particles individually, or negative and positive controls, and then measured for IFN-γ and IL-4 levels. Results showed that both IL-4 (Figure 5A) and IFN-γ responses (Figure 5B) were elicited. To investigate whether the above observed tetravalent immune responses induced by vaccines could confer protection to all DENV serotypes, 3 weeks after the last boost, mice vaccinated with the DDP regimen were challenged i.c. with DENV1, DENV3, or DENV4 separately (Figure 1A). After DENV1 or DENV3 challenge, the mean body weight loss was only 8–11%, statistically lower than that of the controls (*P* < 0.05, Figure 6A,B); after DENV4 challenge, vaccinated mice had a body weight loss of 16%, again significantly lower than that of the controls (35%, *P* < 0.01, Figure 6C). Moreover, a higher survival rate and a prolonged survival time were observed in vaccinated mice (62.5%) than controls (0%, *P* < 0.05, Figure 6D). Taken together, these results demonstrated that the cE80 immunization with the DDP regimen could induce protective immunity to four serotypes of DENV.

**Table 1:** GMTs of DENV-specific IgG and PRNT_50_ in sera of DDP group.

Coating antigens/viruses	Subgroups	IgG (95% CI)	*P* value^a^	PRNT_50_ (95% CI)	*P* value^a^
DENV1	Vaccine	16,890 (6,432–32,176)	***	160 (38–358)	**
	Control	230 (110–393)		10 (10–10)	
DENV2	Vaccine	25,600 (16,906–39,794)	***	422 (251–740)	***
	Control	200 (200–200)		10 (10–10)	
DENV3	Vaccine	6,400 (6,400–6,400)	**	106 (40–201)	**
	Control	230 (110–393)		10 (10–10)	
DENV4	Vaccine	4,850 (1,847–9,240)	**	61 (23–116)	*
	Control	230 (110–393)		10 (10–10)	


^a^P values were analyzed by one-way ANOVA when compared titers in vaccine subgroups with those of control subgroups, *n = 5*, *P < 0.5, **P < 0.01, ***P < 0.001. GMTs, geometric mean titers.

## Discussion

### Heterologous DDP Immunization Regimen Using a Single Consensus-Based cE80 Vaccine Effectively Improved Systemic Immune Responses to DENV2

Ideal dengue vaccines and appropriate vaccination regimens are urgently needed to solve the dengue problem on a global scale. In this study, we first compared three vaccination regimens with a DNA and a protein vaccine containing the same cE80 sequence in either DDD, or DDP, or DPP format, and demonstrated that DDP is the optimal method for inducing robust neutralizing antibodies, strong antigen-specific T cell responses, and effective protection against DENV2.

Consensus sequence coding target antigens has been widely applied in vaccine development to induce universal and specific immune responses, especially for those viruses with high degree of genetic variation, such as influenza virus and HIV (Yan et al., 2014; Yang et al., 2015). Previously, based on the consensus sequences of the ectodomain of E protein (E80) of 3,127 DENV strains including all four DENV serotypes, we designed and constructed a tetravalent subunit vaccine, designated as cE80. As a single immunogen rather than conventional tetravalent formulation with four individual vaccine components, the cE80 might mechanistically avoid either the potential interference among each of the four components, or the immune dominance of a particular antigenic component of a specific serotype. Moreover, it can reduce the cost and complexity for production of a tetravalent vaccine. Indeed, we have shown that homologous immunization with three doses of cE80 protein was sufficient to induce binding and tetravalent nAb and Th2 type immune response (Sun et al., 2017).

DNA vaccine has many advantages such as simplicity of manufacturing, ease of preparation, cost-effectiveness and biological stability (Dhama et al., 2008). In fact, a tetravalent dengue DNA vaccine tested in a phase I clinical trial has demonstrated its safety, tolerability, and capacity of eliciting DENV-specific IFN-γ responses (Danko et al., 2018). We have also demonstrated that DNA vaccine was capable of inducing balanced immune responses and effective protection against DENVs (Zheng et al., 2017).

When the protein and DNA vaccines were tested together, we found that homologous immunization with three doses of pV-cE80 DNA (DDD) and was less effective than heterologous prime-boost regimens (DPP and DDP) using pV-cE80 and cE80 protein in combination. The specific reason for this observation is unclear, but some possibilities could be considered.

For homologous DNA immunization, our previous work showed that 50 μg of each immunogen dose was enough to induce strong immune responses and protection in mice (Wang et al., 2018). However, in this study, immunization with three doses of 50 μg of pV-cE80 DNA only induced modest immune responses and protection, revealed by the limited antibody titers, CD8^+^ T cell response and 75% survival rate after DENV2 challenge, which may due to the mismatch between sequence of cE80 vaccine and challenge virus DENV2. In contrast, for heterologous immunization regimens, mice immunized using the DPP or DDP regimens triggered effective anti-DENV2 humoral and cellular immune responses, as well as cytokine responses and effective protection as compared with DDD. In particular, the DDP immunization regimen induced the strongest immune responses and protection among all vaccination regimens tested (Figure 1–4), indicating obvious advantages of the DDP, due probably to protein boosting of memory T and B cell responses to conserved antigens, elicited by DNA double priming.

Our results are consistent with some similar studies. De Filette et al. (2014) reported that the DNA prime-protein boost immunization strategy could result in a marked increase in nAb titer and a complete protection against a lethal West Nile virus (WNV) infection as compared to injection of E protein of WNV alone. Kadkhodayan et al. (2017) demonstrated that the DNA prime-protein boost regimen significantly improved specific T cell response and antibody response against HIV or influenza virus as compared to DNA or protein vaccination alone. However, previous attempt to induce broad protection against multiple DENV serotypes has encountered some difficulty. Apt et al. (2006) and Raviprakash et al. (2006) developed three recombinant DENV E DNA constructs containing shared epitopes from four DENVs using DNA shuffling and screening technologies, but discovered that homologous vaccination with the shuffled constructs only provided limited protection against either DENV1 or DENV2 in vaccinated animals. Similarly, there was also limited immunogenicity when vaccination with pV-cE80 DNA only in this study. In contrast, heterologous vaccination regimens not only induced high level of memory T cells, but also elevated antibody response and conferred protection against all four serotypes of DENV. Thus, the tactic of DNA prime, followed by protein boost may be suitable for developing DENV vaccine as it did for other vaccines (De Filette et al., 2014; Wang et al., 2017).

It is not completely clear why heterologous vaccination regimen produces a better immune response than homologous immunization. Theoretically, the immune responses elicited by DNA or protein are associated with the route of antigen delivery and the mode by which antigens are presented to B- or T-lymphocytes. DNA vaccines use the host cell machinery for *in vivo* synthesis of target antigens, which can be delivered through intra- or extracellular pathways to trigger both MHC-I or MHC-II restricted T cell immune responses. Moreover, intracellular antigens expressed by DNA vaccine tend to fold in their native conformation and correctly glycosylated form that may display the neutralizing epitopes in a similar pattern as the native virus. In contrast, protein vaccines are delivered exogenously to host cells and they mostly elicit antibody response in connection with the MHC-II pathway of antigen presentation. For these reasons, DNA is often used as a prime antigen for pre-sensitizing antigen-specific memory B cells and increasing the quantity and diversity of CD4^+^ T cell clones, and at the same time it stimulates CD8^+^ T cell activation. Then the protein-boost stimulates memory B cells to divide and differentiate into plasma cells that secret antibody with greater magnitude (Letellier et al., 2008; Ranasinghe and Ramshaw, 2009). Accordingly, the activation of both humoral and cellular immune responses by a DNA, followed by boosting with a protein vaccine in the heterologous vaccination regimen will likely be more effective at inducing protective immunity.

Interestingly, we found that the DDP regimen offered more protective efficacy than the DPP regimen in this study. The mechanisms for this observation can only be speculated at the moment. Because antigen is mostly expressed in local myocytes and/or keratinocytes after DNA vaccination, a single priming with DNA priming in the DPP regimen may present too low an amount of antigen to generate a long-lived antibody-secreting plasma cell population and induce a potent high-affinity antibody response, but should be possible to establish a small number of non-antibody secreting memory B cells. In comparison, for the DDP regimen, after once protein boost as an adjuvant following two DNA immunizations, antibody-secreting memory B cells could be activated, and the recruitment of Langerhans cell from injection sites to draining lymph nodes could be stimulated (Porgador et al., 1998; Kahlon et al., 2003), both of which could contribute to a better induction of antibody responses. These intricate interactions during vaccination process might explain why the DDP regimen led to the induction of higher level of antibody and cytokine responses and conferred more effective protection against DENV2 than the DDD or DPP. Taken together, the results suggested that the heterologous DDP immunization regimen would be advantageous in significantly improving the DENV2-specific CD8^+^ responses and simultaneous Th1 and Th2 responses (Aviles et al., 2015; Wang et al., 2017; Cao et al., 2018; Jung et al., 2018). Therefore, the DDP vaccination regimen would be an appropriate strategy for developing dengue vaccines. Whether the same principle applies to other vaccine antigens needs to be further investigated.

### Tetravalent Immune Responses and Protection Were Elicited by a Single cE80 Vaccine Candidate via the DDP Vaccination Regimen

We also evaluated whether the DDP vaccination regimen could evoke immune responses and protection to other three DENV serotypes. Significantly, the DDP vaccination not only induced significantly levels of Th2 (IL-4) and Th1 (IFN-γ) cytokine responses upon *in vitro* stimulation with purified DENV1, or DENV2, or DENV3 or DENV4 particles (Figure 5), high levels of IgG and nAb responses against four DENV serotypes, but also protections against DENV1, DENV3 and DENV4 (Figure 6). In this study, body weight changes were used for evaluating *in vivo* protective efficacy in mice challenged with DENV1 or DENV3. Only 8–11% of body weight loss was observed after DENV1 and DENV3 challenge in vaccinated group mice, but 18–24% of body weight loss in controls. In case of challenge by DENV4, the DDP immunization provided 62.5% survival rate and significantly less body weight loss than controls. In combination with the results of DENV2, above results indicated that the cE80 vaccines used in a DDP immunization regimen could induce tetravalent protection against infection by all four DENV serotypes. Therefore, it represents a promising immunization strategy for developing dengue vaccine.

There are some limitations in this study. Firstly, among four serotypes of DENV, there was a relatively preponderant immune response to DENV2 as revealed by the highest titers of IgG and nAb (Table 1). This may be related to the fact that the cE80 sequence shares the highest homology in amino acid DENV2, and it has the shortest evolutionary distance from DENV2, and phylogenetically clustered together with DENV2 (Sun et al., 2017); secondly, cE80 contains many predicted epitopes within DENV2 (Supplementary Figure S1 and Table S1). These factors could have contributed to a more robust humoral immune response and protective immunity to DENV (Schein et al., 2012). Additionally, there is a growing consensus that CD8^+^ T cell immune response plays an important role in the protection induced by dengue vaccine (Lam et al., 2017). It was reported that a stronger CD8^+^ T cell response requires non-structural protein (NS), although there are some CD8^+^ T cell epitopes within E protein (Yauch et al., 2009). The lack of NS proteins is also a flaw for our recombinant cE80 vaccine candidate. In further studies, modification of vaccine design by including *NS* gene(s) might improve the cellular immune response and the balance among the tetravalent immune responses induced by a single dengue vaccine.

Although the development in dengue DNA vaccine has not been problem-free, recent studies have indicated that Zika DNA vaccines are highly effective at generating robust humoral and cellular immune responses in mice and monkeys (Dowd et al., 2016; Hampton, 2016; Griffin et al., 2017). Because of the structural and genetic closeness between ZIKV and DENV, the success of Zika DNA vaccines will likely to inform the rational development of dengue DNA vaccine.

## Conclusion

As an extension of our previous work with cE80 protein (P) vaccine, this study examined whether adding a DNA vaccine (D) encoding the same antigen, in three vaccination regiments: DDD, DDP, and DPP, will augment the efficacy of the cE80 vaccine. We found that, among the three DNA-base immunization regimens, the DDP regimen is the optimal regimen which enabled the generation of both strong and relatively balanced humoral and cellular immune responses to all four serotypes of DENV, as well as desirable cytokine response. Importantly, our vaccine candidate when used in DDP regimen, afforded effective protection against the challenge by each of the four serotypes of DENV. Our results, for the first time, demonstrated vaccine with a single dengue virus derived sequence conferring tetravalent protection in mice. Our results offer a prototype DENV vaccine that could be improved, and then tested in non-human primate model, and eventually in human clinical trials.

## Ethics Statement

The animal experiments were performed according to “Regulations for the Administration of Affairs Concerning Experimental Animals” which is the national guidelines for the care and use of animals in scientific research. All experimental procedures were approved by the Institutional Animal Care and Use Committee of Capital Medical University, China.

## Author Contributions

RW designed and performed the experiments, analyzed the data, and wrote the manuscript. XZ, JS, KF, NG, and DF helped the experimental design. XJ designed the codon-optimized sequence. HC designed the research, analyzed the data, and wrote the manuscript. JA designed the principal research, supervised the project, and drafted the manuscript.

## Conflict of Interest Statement

The authors declare that the research was conducted in the absence of any commercial or financial relationships that could be construed as a potential conflict of interest.

## Supplementary Material

FIGURE S1 Characterization of amino acid residues and *in vitro* expression of cE80. **(A)** Distribution of predicted amino acid residues along the cE80 sequence aligned with DENV1–4 E80 proteins. Line 1 (number), residue positions; Line 2 (red rhombuses), predicted protein binding region; Line 3 (dark red and blue bars), dark red bar represents helix and blue bar represents strand; Line 4 (blue, white and yellow bars), blue bar represents the region exposed on the surface, white and yellow bars represent intermediate and buried regions; Line 5 (green bar), disordered regions. The predicted features were created by an online website (http://open.predictprotein.org/). **(B)** *In vitro* expression of cE80. BHK-21 cells were transfected with recombinant plasmid pV-cE80. Transient expression of cE80 protein was examined by indirect immunofluorescence assay. After fixation with paraformaldehyde, the cells were permeabilized with 0.3% Triton X-100 in phosphate buffer solution and then blocked with bovine serum albumin. Immune sera obtained from mice infected with DENV1, 2, 3, or 4 were used as the primary antibodies. Cells were stained with goat-anti-mouse IgG-FITC (in green) and counterstained with DAPI (in blue) to identify cell nuclei (×200). The white bar represents 50 μm.

FIGURE S2 Gating strategy for T cell subsets in splenocytes. Cells stained with different combination of antibodies were sequentially gated. First, gated on the life cells (FSC area vs. SSC area, left). Second, gated on single cells (FSC width vs. FSC area, middle). Finally, gated on CD3^+^ CD8^+^ T cell (right).

TABLE S1 Homologous alignment of *cE80* with different serotypes of DENV at nucleotide and amino acid level. DENV, dengue virus; AA, amino acid. ^a^Standard strains.

TABLE S2 Immunization groups and schedule. i.m., intramuscularly; s.c., subcutaneously; EP, electroporation.

TEXT S1 Sequence of *cE80.*

## References

[B1] Aggarwal A. Garg N. (2017). Newer vaccines against mosquito-borne diseases. *Indian J. Pediatr.* 85 117–123. 10.1007/s12098-017-2383-4 28560654

[B2] Apt D. Raviprakash K. Brinkman A. Semyonov A. Yang S. Skinner C. (2006). Tetravalent neutralizing antibody response against four dengue serotypes by a single chimeric dengue envelope antigen. *Vaccine* 24 335–344. 10.1016/j.vaccine.2005.07.100 16125280

[B3] Aviles J. Bello A. Wong G. Fausther-Bovendo H. Qiu X. Kobinger G. (2015). Optimization of prime-boost vaccination strategies against mouse-adapted ebolavirus in a short-term protection study. *J. Infect. Dis.* 212(Suppl. 2), S389–S397. 10.1093/infdis/jiv175 26038398

[B4] Beltramello M. Williams K. L. Simmons C. P. Macagno A. Simonelli L. Quyen N. T. (2010). The human immune response to Dengue virus is dominated by highly cross-reactive antibodies endowed with neutralizing and enhancing activity. *Cell Host Microbe* 8 271–283. 10.1016/j.chom.2010.08.007 20833378 PMC3884547

[B5] Cao W. Mishina M. Amoah S. Mboko W. P. Bohannon C. McCoy J. (2018). Nasal delivery of H5N1 avian influenza vaccine formulated with GenJet or in vivo-jetPEI((R)) induces enhanced serological, cellular and protective immune responses. *Drug Deliv.* 25 773–779. 10.1080/10717544.2018.1450909 29542358 PMC6058713

[B6] Carter D. M. Darby C. A. Lefoley B. C. Crevar C. J. Alefantis T. Oomen R. (2016). Design and characterization of a computationally optimized broadly reactive hemagglutinin vaccine for H1N1 influenza viruses. *J. Virol.* 90 4720–4734. 10.1128/JVI.03152-15 26912624 PMC4836330

[B7] Danko J. R. Kochel T. Teneza-Mora N. Luke T. C. Raviprakash K. Sun P. (2018). Safety and immunogenicity of a tetravalent dengue DNA vaccine administered with a cationic lipid-based adjuvant in a phase 1 clinical trial. *Am. J. Trop. Med. Hyg.* 98 849–856. 10.4269/ajtmh.17-0416 29363446 PMC5930886

[B8] De Filette M. Soehle S. Ulbert S. Richner J. Diamond M. S. Sinigaglia A. (2014). Vaccination of mice using the west nile virus E-protein in a DNA prime-protein boost strategy stimulates cell-mediated immunity and protects mice against a lethal challenge. *PLoS One* 9:e87837. 10.1371/journal.pone.0087837 24503579 PMC3913677

[B9] de Soarez P. C. Silva A. B. Randi B. A. Azevedo L. M. Novaes H. M. D. Sartori A. M. C. (2019). Systematic review of health economic evaluation studies of dengue vaccines. *Vaccine* 37 2298–2310. 10.1016/j.vaccine.2019.03.026 30910406

[B10] Dhama K. Mahendran M. Gupta P. K. Rai A. (2008). DNA vaccines and their applications in veterinary practice: current perspectives. *Vet. Res. Commun.* 32 341–356. 10.1007/s11259-008-9040-3 18425596 PMC7089108

[B11] Dowd K. A. Ko S. Y. Morabito K. M. Yang E. S. Pelc R. S. DeMaso C. R. (2016). Rapid development of a DNA vaccine for Zika virus. *Science* 354 237–240. 27708058 10.1126/science.aai9137 PMC5304212

[B12] Ferguson N. M. Rodriguez-Barraquer I. Dorigatti I. Mier Y. T.-R. L. Laydon D. J. Cummings D. A. (2016). Benefits and risks of the sanofi-pasteur dengue vaccine: modeling optimal deployment. *Science* 353 1033–1036. 10.1126/science.aaf9590 27701113 PMC5268127

[B13] George S. L. Wong M. A. Dube T. J. Boroughs K. L. Stovall J. L. Luy B. E. (2015). Safety and immunogenicity of a live attenuated tetravalent dengue vaccine candidate in flavivirus-naive adults: a randomized, double-blinded phase 1 clinical trial. *J. Infect. Dis.* 212 1032–1041. 10.1093/infdis/jiv179 25791116 PMC4559193

[B14] Germann T. Bongartz M. Dlugonska H. Hess H. Schmitt E. Kolbe L. (1995). Interleukin-12 profoundly up-regulates the synthesis of antigen-specific complement-fixing IgG2a, IgG2b and IgG3 antibody subclasses in vivo. *Eur. J. Immunol.* 25 823–829. 10.1002/eji.1830250329 7705414

[B15] Griffin B. D. Muthumani K. Warner B. M. Majer A. Hagan M. Audet J. (2017). DNA vaccination protects mice against Zika virus-induced damage to the testes. *Nat. Commun.* 8:15743. 10.1038/ncomms15743 28589934 PMC5467228

[B16] Hampton T. (2016). DNA vaccine protects monkeys against zika virus infection. *JAMA* 316:1755. 10.1001/jama.2016.15862 27802535

[B17] Henein S. Swanstrom J. Byers A. M. Moser J. M. Shaik S. F. Bonaparte M. (2017). Dissecting antibodies induced by a chimeric yellow fever-dengue, live-attenuated, tetravalent dengue vaccine (CYD-TDV) in naive and dengue-exposed individuals. *J. Infect. Dis.* 215 351–358. 10.1093/infdis/jiw576 27932620 PMC6392503

[B18] Jarvi S. I. Hu D. Misajon K. Coller B. A. Wong T. Lieberman M. M. (2013). Vaccination of captive nene (Branta sandvicensis) against west nile virus using a protein-based vaccine (WN-80E). *J. Wildl. Dis.* 49 152–156. 10.7589/2011-12-363 23307381

[B19] Jung S. Y. Kang K. W. Lee E. Y. Seo D. W. Kim H. L. Kim H. (2018). Heterologous prime-boost vaccination with adenoviral vector and protein nanoparticles induces both Th1 and Th2 responses against middle east respiratory syndrome coronavirus. *Vaccine* 36 3468–3476. 10.1016/j.vaccine.2018.04.082 29739720 PMC7115429

[B20] Kadkhodayan S. Jafarzade B. S. Sadat S. M. Motevalli F. Agi E. Bolhassani A. (2017). Combination of cell penetrating peptides and heterologous DNA prime/protein boost strategy enhances immune responses against HIV-1 Nef antigen in BALB/c mouse model. *Immunol. Lett.* 188 38–45. 10.1016/j.imlet.2017.06.003 28602843

[B21] Kahlon R. Hu Y. Orteu C. H. Kifayet A. Trudeau J. D. Tan R. (2003). Optimization of epicutaneous immunization for the induction of CTL. *Vaccine* 21 2890–2899. 10.1016/s0264-410x(03)00141-5 12798632

[B22] Kirkpatrick B. D. Whitehead S. S. Pierce K. K. Tibery C. M. Grier P. L. Hynes N. A. (2016). The live attenuated dengue vaccine TV003 elicits complete protection against dengue in a human challenge model. *Sci. Transl. Med.* 8:330ra336. 10.1126/scitranslmed.aaf1517 27089205

[B23] Lam J. H. Chua Y. L. Lee P. X. Martinez Gomez J. M. Ooi E. E. Alonso S. (2017). Dengue vaccine-induced CD8+ T cell immunity confers protection in the context of enhancing, interfering maternal antibodies. *JCI Insight* 2:94500. 10.1172/jci.insight.94500 29263304 PMC5752305

[B24] Lazear H. M. Diamond M. S. (2016). Zika virus: new clinical syndromes and its emergence in the Western Hemisphere. *J. Virol.* 90 4864–4875. 10.1128/JVI.00252-16 26962217 PMC4859708

[B25] Letellier C. Boxus M. Rosar L. Toussaint J. F. Walravens K. Roels S. (2008). Vaccination of calves using the BRSV nucleocapsid protein in a DNA prime-protein boost strategy stimulates cell-mediated immunity and protects the lungs against BRSV replication and pathology. *Vaccine* 26 4840–4848. 10.1016/j.vaccine.2008.06.100 18644416 PMC7115630

[B26] Mallios R. R. (2003). A consensus strategy for combining HLA-DR binding algorithms. *Hum. Immunol.* 64 852–856. 10.1016/s0198-8859(03)00142-3 12941539

[B27] Meyerhoff R. R. Scearce R. M. Ogburn D. F. Lockwood B. Pickeral J. Kuraoka M. (2017). HIV-1 consensus envelope-induced broadly binding antibodies. *AIDS Res. Hum. Retroviruses* 33 859–868. 10.1089/AID.2016.0294 28314374 PMC5564029

[B28] Miksanek Z. Wolford R. (2017). *Dengue Fever.* Treasure Island, FL: StatPearls.

[B29] Moutaftsi M. Peters B. Pasquetto V. Tscharke D. C. Sidney J. Bui H. H. (2006). A consensus epitope prediction approach identifies the breadth of murine T(CD8+)-cell responses to vaccinia virus. *Nat. Biotechnol.* 24 817–819. 10.1038/nbt1215 16767078

[B30] Porgador A. Irvine K. R. Iwasaki A. Barber B. H. Restifo N. P. Germain R. N. (1998). Predominant role for directly transfected dendritic cells in antigen presentation to CD8+ T cells after gene gun immunization. *J. Exp. Med.* 188 1075–1082. 10.1084/jem.188.6.1075 9743526 PMC2212529

[B31] Ranasinghe C. Ramshaw I. A. (2009). Genetic heterologous prime-boost vaccination strategies for improved systemic and mucosal immunity. *Expert Rev. Vaccines* 8 1171–1181. 10.1586/erv.09.86 19722891

[B32] Raviprakash K. Apt D. Brinkman A. Skinner C. Yang S. Dawes G. (2006). A chimeric tetravalent dengue DNA vaccine elicits neutralizing antibody to all four virus serotypes in rhesus macaques. *Virology* 353 166–173. 10.1016/j.virol.2006.05.005 16814355

[B33] Rupp R. Luckasen G. J. Kirstein J. L. Osorio J. E. Santangelo J. D. Raanan M. (2015). Safety and immunogenicity of different doses and schedules of a live attenuated tetravalent dengue vaccine (TDV) in healthy adults: a phase 1b randomized study. *Vaccine* 33 6351–6359. 10.1016/j.vaccine.2015.09.008 26384447

[B34] Saez-Llorens X. Tricou V. Yu D. Rivera L. Jimeno J. Villarreal A. C. (2018). Immunogenicity and safety of one versus two doses of tetravalent dengue vaccine in healthy children aged 2-17 years in Asia and Latin America: 18-month interim data from a phase 2, randomised, placebo-controlled study. *Lancet Infect. Dis.* 18 162–170. 10.1016/S1473-3099(17)30632-1 29122463

[B35] Saez-Llorens X. Tricou V. Yu D. Rivera L. Tuboi S. Garbes P. (2017). Safety and immunogenicity of one versus two doses of Takeda’s tetravalent dengue vaccine in children in Asia and Latin America: interim results from a phase 2, randomised, placebo-controlled study. *Lancet Infect. Dis.* 17 615–625. 10.1016/S1473-3099(17)30166-4 28365225

[B36] Schein C. H. Bowen D. M. Lewis J. A. Choi K. Paul A. van der Heden van Noort G. J. (2012). Physicochemical property consensus sequences for functional analysis, design of multivalent antigens and targeted antivirals. *BMC Bioinformatics* 13(Suppl. 13):S9. 10.1186/1471-2105-13-S13-S9 23320474 PMC3426803

[B37] Sette A. Fikes J. (2003). Epitope-based vaccines: an update on epitope identification, vaccine design and delivery. *Curr. Opin. Immunol.* 15 461–470. 10.1016/s0952-7915(03)00083-9 12900280

[B38] Sette A. Livingston B. McKinney D. Appella E. Fikes J. Sidney J. (2001). The development of multi-epitope vaccines: epitope identification, vaccine design and clinical evaluation. *Biologicals* 29 271–276. 10.1006/biol.2001.0297 11851327

[B39] Sirivichayakul C. Barranco-Santana E. A. Esquilin-Rivera I. Oh H. M. Raanan M. Sariol C. A. (2016). Safety and immunogenicity of a tetravalent dengue vaccine candidate in healthy children and adults in dengue-endemic regions: a randomized, placebo-controlled phase 2 study. *J. Infect. Dis.* 213 1562–1572. 10.1093/infdis/jiv762 26704612

[B40] Sridhar S. Luedtke A. Langevin E. Zhu M. Bonaparte M. Machabert T. (2018). Effect of dengue serostatus on dengue vaccine safety and efficacy. *N. Engl. J. Med.* 379 327–340. 10.1056/NEJMoa1800820 29897841

[B41] Stanaway J. D. Shepard D. S. Undurraga E. A. Halasa Y. A. Coffeng L. E. Brady O. J. (2016). The global burden of dengue: an analysis from the global burden of disease study 2013. *Lancet Infect. Dis.* 16 712–723. 10.1016/S1473-3099(16)00026-8 26874619 PMC5012511

[B42] Sun J. Li M. Wang Y. Hao P. Jin X. (2017). Elaboration of tetravalent antibody responses against dengue viruses using a subunit vaccine comprised of a single consensus dengue envelope sequence. *Vaccine* 35 6308–6320. 10.1016/j.vaccine.2017.09.063 28987441

[B43] Trent D. W. (1977). Antigenic characterization of flavivirus structural proteins separated by isoelectric focusing. *J. Virol.* 22 608–618. 69040 10.1128/jvi.22.3.608-618.1977 PMC515759

[B44] Wang G. Yin R. Zhou P. Ding Z. (2017). Combination of the immunization with the sequence close to the consensus sequence and two DNA prime plus one VLP boost generate H5 hemagglutinin specific broad neutralizing antibodies. *PLoS One* 12:e0176854. 10.1371/journal.pone.0176854 28542275 PMC5443486

[B45] Wang R. Liao X. Fan D. Wang L. Song J. Feng K. (2018). Maternal immunization with a DNA vaccine candidate elicits specific passive protection against post-natal Zika virus infection in immunocompetent BALB/c mice. *Vaccine* 36 3522–3532. 10.1016/j.vaccine.2018.04.051 29753607

[B46] Whitehead S. S. Durbin A. P. Pierce K. K. Elwood D. McElvany B. D. Fraser E. A. (2017). In a randomized trial, the live attenuated tetravalent dengue vaccine TV003 is well-tolerated and highly immunogenic in subjects with flavivirus exposure prior to vaccination. *PLoS Negl. Trop. Dis.* 11:e0005584. 10.1371/journal.pntd.0005584 28481883 PMC5436874

[B47] WHO Report (2018). Dengue vaccine: WHO position paper, September 2018 - Recommendations. *Vaccine* 10.1016/j.vaccine.2018.09.063 [Epub ahead of print]. 30424888

[B48] Wilder-Smith A. Hombach J. Ferguson N. Selgelid M. O’Brien K. Vannice K. (2019). Deliberations of the strategic advisory group of experts on immunization on the use of CYD-TDV dengue vaccine. *Lancet Infect. Dis.* 19 e31–e38. 10.1016/S1473-3099(18)30494-8 30195995

[B49] Yan J. Villarreal D. O. Racine T. Chu J. S. Walters J. N. Morrow M. P. (2014). Protective immunity to H7N9 influenza viruses elicited by synthetic DNA vaccine. *Vaccine* 32 2833–2842. 10.1016/j.vaccine.2014.02.038 24631084 PMC4221260

[B50] Yan J. Yoon H. Kumar S. Ramanathan M. P. Corbitt N. Kutzler M. (2007). Enhanced cellular immune responses elicited by an engineered HIV-1 subtype B consensus-based envelope DNA vaccine. *Mol. Ther.* 15 411–421. 10.1038/sj.mt.6300036 17235321

[B51] Yang O. O. Ali A. Kasahara N. Faure-Kumar E. Bae J. Y. Picker L. J. (2015). Short conserved sequences of HIV-1 are highly immunogenic and shift immunodominance. *J. Virol.* 89 1195–1204. 10.1128/JVI.02370-14 25378501 PMC4300636

[B52] Yauch L. E. Zellweger R. M. Kotturi M. F. Qutubuddin A. Sidney J. Peters B. (2009). A protective role for dengue virus-specific CD8+ T cells. *J. Immunol.* 182 4865–4873. 10.4049/jimmunol.0801974 19342665 PMC2674070

[B53] Zheng X. Chen H. Wang R. Fan D. Feng K. Gao N. (2017). Effective protection induced by a monovalent DNA vaccine against dengue virus (DV) serotype 1 and a bivalent DNA vaccine against DV1 and DV2 in Mice. *Front. Cell Infect. Microbiol.* 7:175. 10.3389/fcimb.2017.00175 28553618 PMC5427067

