# Supplementary figures and images for: Vaccination With a Single Consensus Envelope Protein Ectodomain Sequence Administered in a Heterologous Regimen Induces Tetravalent Immune Responses and Protection Against Dengue Viruses in Mice
Source: Front Microbiol. 2019 May 10;10:1113. doi: 10.3389/fmicb.2019.01113 (PMC6524413; doi:10.3389/fmicb.2019.01113)

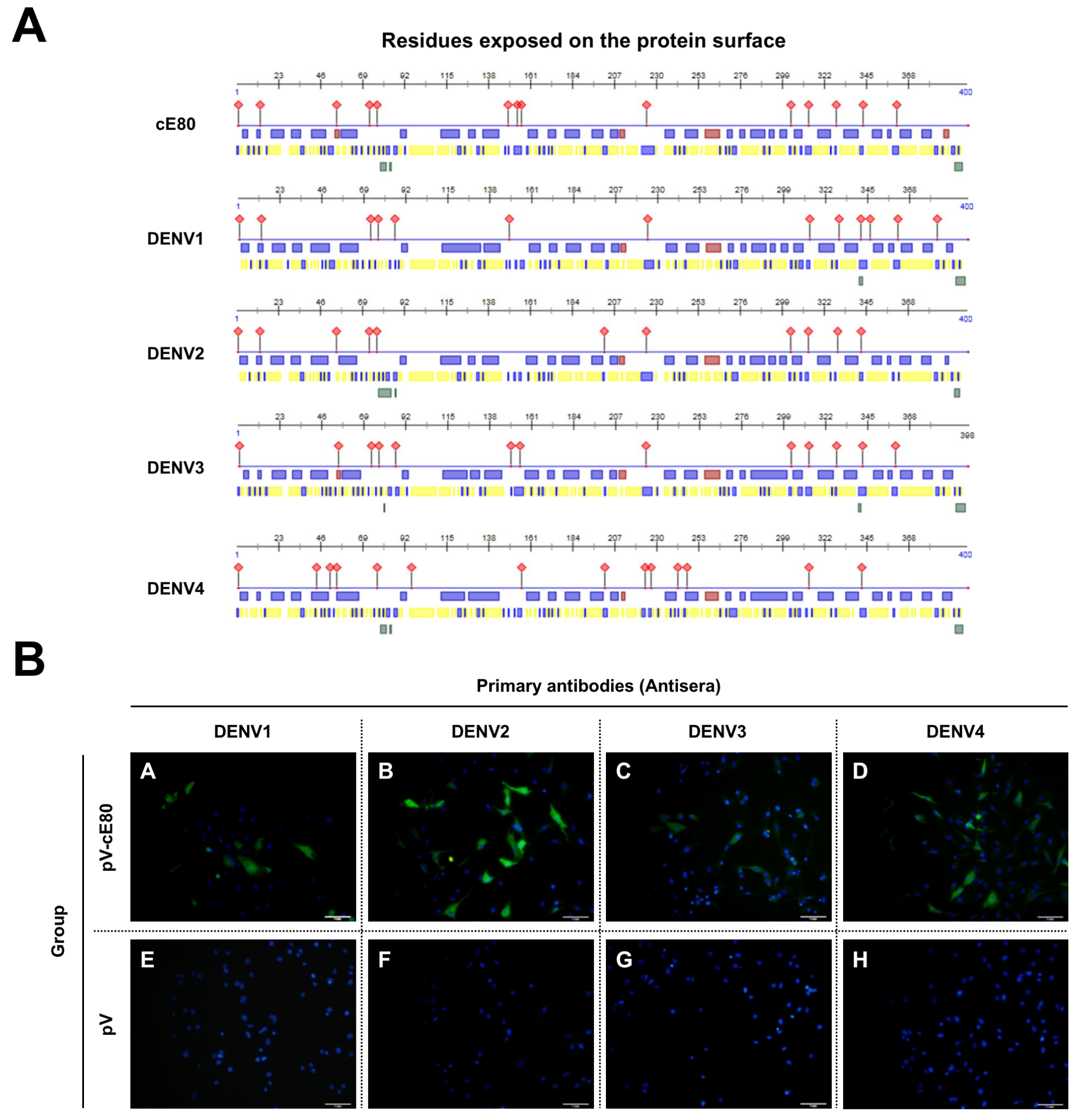

Supplement: FIGURE S1 — Characterization of amino acid residues and in vitro expression of cE80. (A) Distribution of predicted amino acid residues along the cE80 sequence aligned with DENV1–4 E80 proteins. Line 1 (number), residue positions; Line 2 (red rhombuses), predicted protein binding region; Line 3 (dark red and blue bars), dark red bar represents helix and blue bar represents strand; Line 4 (blue, white and yellow bars), blue bar represents the region exposed on the surface, white and yellow bars represent intermediate and buried regions; Line 5 (green bar), disordered regions. The predicted features were created by an online website (http://open.predictprotein.org/). (B) In vitro expression of cE80. BHK-21 cells were transfected with recombinant plasmid pV-cE80. Transient expression of cE80 protein was examined by indirect immunofluorescence assay. After fixation with paraformaldehyde, the cells were permeabilized with 0.3% Triton X-100 in phosphate buffer solution and then blocked with bovine serum albumin. Immune sera obtained from mice infected with DENV1, 2, 3, or 4 were used as the primary antibodies. Cells were stained with goat-anti-mouse IgG-FITC (in green) and counterstained with DAPI (in blue) to identify cell nuclei (×200). The white bar represents 50 μm. [file Image_1.TIFF]

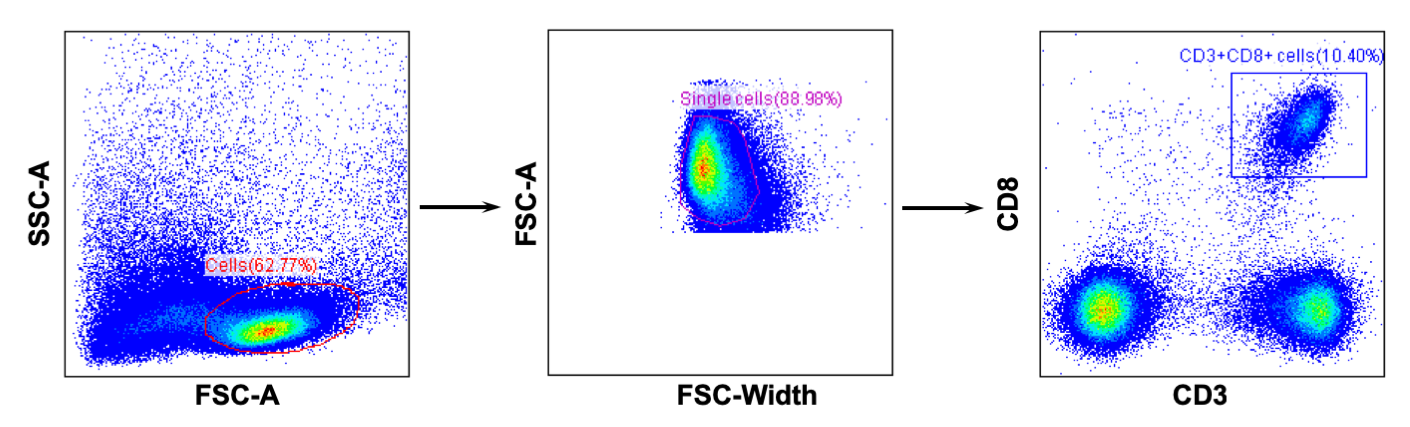

Supplement: FIGURE S2 — Gating strategy for T cell subsets in splenocytes. Cells stained with different combination of antibodies were sequentially gated. First, gated on the life cells (FSC area vs. SSC area, left). Second, gated on single cells (FSC width vs. FSC area, middle). Finally, gated on CD3+ CD8+ T cell (right). [file Image_2.PNG]
