# Supplementary material for: Vaccination With a Single Consensus Envelope Protein Ectodomain Sequence Administered in a Heterologous Regimen Induces Tetravalent Immune Responses and Protection Against Dengue Viruses in Mice
Source: Front Microbiol. 2019 May 10;10:1113. doi: 10.3389/fmicb.2019.01113 (PMC6524413; doi:10.3389/fmicb.2019.01113)
Supplement: TABLE S1 — Homologous alignment of cE80 with different serotypes of DENV at nucleotide and amino acid level. DENV, dengue virus; AA, amino acid. aStandard strains. [file Table_1.doc]

| Supplementary Table 1  Homologous alignment of *cE80* with different serotypes of DENV at nucleotide and amino acid level | | | | | | | | | | | | | | |
| --- | --- | --- | --- | --- | --- | --- | --- | --- | --- | --- | --- | --- | --- | --- |
| DENV1 | | |  | DENV2 | | |  | DENV3 | | |  | DENV4 | | |
| Strain | DNA (%) | AA (%) |  | Strain | DNA (%) | AA (%) |  | Strain | DNA (%) | AA (%) |  | Strain | DNA (%) | AA (%) |
| Hawaii^a^ | 87.41 | 73.33 |  | New Guinea C^a^ | 92.33 | 87.13 |  | H87^a^ | 90.05 | 75.87 |  | H241^a^ | 90.05 | 75.87 |
| Okinawa | 89.75 | 75.75 |  | D83-307 | 94.50 | 88.25 |  | 13/GZ/26547 | 90.75 | 77.25 |  | SG(EHI)D4/30313Y13 | 90.75 | 77.25 |
| Khabar2984 | 89.03 | 74.31 |  | 23085/1960/Vellore | 94.25 | 88.25 |  | D3BR/CU6/02 | 90.82 | 77.17 |  | NIV_62235 | 90.82 | 77.17 |
| GD1991 | 89.75 | 75.50 |  | ThNH81/93 | 92.86 | 86.21 |  | NC89/060289-283 | 90.50 | 77.00 |  | D4/IDN/Bali_069/2011 | 90.50 | 77.00 |
| D1/SBY21/12 | 89.75 | 75.25 |  | 1008-DHF-11/28/2001 | 91.85 | 85.68 |  | D3/Pakistan/2014/I5 | 92.29 | 78.36 |  | D4/VN/Hue422/2013 | 92.29 | 78.36 |
| Abbreviation: DENV, dengue virus; AA, amino acid.  ^a^ Standard strains. | | | | | | | | | | | | | | |
