# Supplementary material for: Vaccination With a Single Consensus Envelope Protein Ectodomain Sequence Administered in a Heterologous Regimen Induces Tetravalent Immune Responses and Protection Against Dengue Viruses in Mice
Source: Front Microbiol. 2019 May 10;10:1113. doi: 10.3389/fmicb.2019.01113 (PMC6524413; doi:10.3389/fmicb.2019.01113)
Supplement: TABLE S2 — Immunization groups and schedule. i.m., intramuscularly; s.c., subcutaneously; EP, electroporation. [file Table_2.doc]

| **Supplementary Table 2**  Immunization groups and schedule | | | | |
| --- | --- | --- | --- | --- |
| Groups | Subgroups | Prime (Dose, Route) | 1^st^ Boost (Dose, Route) | 2^nd^ Boost (Dose, Route) |
| DDD | Vaccine | pV-cE80 (50 μg, i.m.+EP) | pV-cE80 (50 μg, i.m.+EP) | pV-cE80 (50 μg, i.m.+EP) |
|  | Control | pV (50 μg, i.m.+EP) | pV (50 μg, i.m.+EP) | pV (50 μg, i.m.+EP) |
| DPP | Vaccine | pV-cE80 (50 μg, i.m.+EP) | cE80 (10 μg, s.c.) | cE80 (10 μg, s.c.) |
|  | Control | pV (50 μg, i.m.+EP) | Alhydrogel® (10 μl, s.c.) | Alhydrogel® (10 μl, s.c.) |
| DDP | Vaccine | pV-cE80 (50 μg, i.m.+EP) | pV-cE80 (50 μg, i.m.+EP) | cE80 (10 μg, s.c.) |
|  | Control | pV (50 μg, i.m.+EP) | pV (50 μg, i.m.+EP) | Alhydrogel® (10 μl, s.c.) |
| Abbreviations: i.m., intramuscularly; s.c., subcutaneously; EP, electroporation | | | | |
