# Supplementary material for: Vaccination With a Single Consensus Envelope Protein Ectodomain Sequence Administered in a Heterologous Regimen Induces Tetravalent Immune Responses and Protection Against Dengue Viruses in Mice
Source: Front Microbiol. 2019 May 10;10:1113. doi: 10.3389/fmicb.2019.01113 (PMC6524413; doi:10.3389/fmicb.2019.01113)
Supplement: TEXT S1 — Sequence of cE80. [file Data_Sheet_1.doc]

**Supplementary Text 1**

Sequence of *cE80*

ATGCGTTGCGTTGGTATCTCGAACCGCGATTTCGTTGAAGGATTGTCGGGTGCTACTTGGGTGGATGTGGTGCTG

GAACACGGCTCATGCGTCACCACTATGGCCAAGAACAAGCCCACCTTGGACATCGAGCTGATCAAGACAGAAGCT

AAGCAGCCTGCCACCCTCCGTAAGTTGTGCATCGAGGCCAAGCTCACTAACACAACCACTGATTCTCGCTGTCCC

ACACAGGGTGAAGCTAGCCTGAACGAGGAACAAGACAAGAGGTTCGTCTGCAAGCACACCATGGTTGATAGAGGT

TGGGGCAACGGATGTGGTCTGTTCGGCAAGGGATCCCTCGTGACATGCGCTAAGTTCACCTGTAAGAAGAACATG

GAGGGCAAGGTGGTCCAGCCTGAAAACCTGAAGTACACCGTTGTGATCACTGTTCACACAGGCGAGCAGCACCAA

GTGGGAAACGACACCGGAAAGCACGGCAAGGAAATCAAGATCACTCCACAAGCCTCCATCACTGAGGCTGAATTG

ACAGGTTACGGCACACTGACCCTCGAGTGCTCTCCGAGGACAGGACTGGACTTCAACGAAATGGTCCTGCTCACT

ATGAAGAACAAGGCCTGGCTGGTTCACAGACAGTGGTTCTTGGACCTGCCACTCCCATGGCTCTCGGGTGCTGAT

ACCCAGGGCTCCAACTGGATCCAAAAGGAGTTGCTGGTCACTTTCAAGAACGCTCACGCCAAGAAGCAGGAGGTC

GTGGTGCTGGGCAGCCAAGAAGGAGCTATGCACACCGCCCTGACTGGTGCTACAGAGATCCAGATGTCCTCTGGT

ACCCTCTTGTTCGCCGGCCACCTCAAGTGCAGGTTGAAGATGGACAAGTTGCAACTGAAGGGAATGAGCTACTCA

ATGTGCACTGGCAAGTTCAAGCTCGTGAAGGAGATCGCTGAAACCCAGCACGGCACTATCTTGATCCGCGTCCAA

TACGAGGGAGACGATTCTCCCTGCAAGATCCCTTTCGAGATCATGGACCTGGAAGGTCGTCACGTGCTGGGTCGC

CTCATCACCGCCAACCCAATCGTGACTGAGAAGGATTCACCGGTCAACATCGAGGCTGAACCCCCTTTCGGCGAA

TCGTACATCATCATCGGAGTGGGTCCAGGCCAGCTCAAGCTCAACTGGTTCAAGAAGGGTTCGTCCATCGGAAAG
